# Fear of Recurrence in Young Adult Cancer Patients—A Network Analysis

**DOI:** 10.3390/cancers14092092

**Published:** 2022-04-22

**Authors:** Diana Richter, Katharina Clever, Anja Mehnert-Theuerkauf, Antje Schönfelder

**Affiliations:** 1 Department of Medical Psychology and Medical Sociology, University Medical Center Leipzig, 04103 Leipzig, Germany; anja.mehnert@medizin.uni-leipzig.de (A.M.-T.); antje.schoenfelder@medizin.uni-leipzig.de (A.S.); 2 Department of Psychosomatics and Psychotherapy, MEDIAN Centre for Rehabilitation Schmannewitz, 04774 Dahlen, Germany; katharina.clever@median-kliniken.de

**Keywords:** AYA, fear of cancer recurrence, psychosocial, cancer, adolescents and young adults, network analysis

## Abstract

**Simple Summary:**

Fear of cancer recurrence is a main concern for the majority of cancer patients during their disease. Young adults with cancer may experience fear of recurrence throughout their lives, given their relatively long potential survival time. More research is needed to identify evidence-based interventions that can adequately address this fear. Investigating the underlying mechanisms that trigger and sustain fear of cancer recurrence is an important step toward this goal. Network analysis is a useful tool to study symptoms and their structural relationships. The aim of this study is to apply the network analysis approach in a sample of young cancer patients to comprehend their specific symptomatology and define the optimal structure of a questionnaire to assess fear of recurrence in this patient group. Future studies may seek to replicate our findings among different age group samples to identify network structures and potential targets for clinical intervention.

**Abstract:**

Due to the high survival rates of many young cancer patients and a high risk of second tumors, fear of cancer recurrence (FCR) can cause serious impairment for adolescent and young adult (AYA) cancer patients. The aim of this study is to analyze the structure of the Fear of Disease Progression Questionnaire (FoP-Q-12) to better understand the construct of FCR. We performed a cross-sectional survey on a sample of AYA patients aged 15–39 years with different tumor entities. FCR was measured using the FoP-Q-12, and a network analysis was conducted to examine the relationship of FCR symptoms. The importance of individual items in the network was determined using centrality analyses. A total of 247 AYA patients (81.8% female, median age 31.0 years) participated in the study. The mean FCR score in the sample was 35.9 (SD = 9.9). The majority of patients reported having high FCR (59.5%), according to the established cut-off. The two questionnaire items with the strongest association related to fears about work, and the most central symptom was the fear of serious medical interventions. The centrality of emotional issues in the sample indicates that these symptoms should be prioritized in the development of interventions targeting FCR. Further research should address this topic with larger samples of patients in other age groups and in longitudinal studies.

## 1. Introduction

Cancer is associated with significant physical and psychological stress. Fear of cancer recurrence (FCR) is a form of psychological stress that occurs in varying degrees for the majority of cancer patients [1]. FCR is characterized by the fear that the disease may progress or recur. Two primary definitions of this phenomenon have been established, which differ according to the specific fears of “recurrence” and “progression” of the disease [2,3]. Both definitions state that FCR “is a reactive, consciously perceived fear arising from the real-life experience of a serious, potentially life-threatening or disabling illness and its treatment” [4]. Due to the similar relevant features of both constructs, they are used synonymously in this paper. The severity of FCR is defined along a continuum ranging from functional to dysfunctional [4]. FCR is a normal part of coping with disease and may serve a protective function. For example, high FCR may encourage patients to attend regular follow-up examinations or adopt appropriate health behaviors to minimize the risk of cancer recurrence [5,6]. In contrast, many studies have found a significant relationship between high FCR and low quality of life, psychological comorbidities, and heightened distress and physical limitations [7,8,9].

For adolescent and young adult cancer patients (AYA) aged 15–39 years, FCR represents a non-negligible psychological impairment [10]. These young patients likely have a long survival time; however, they are also at high risk for long-term physical and psychological consequences [11,12,13,14]. Studies have shown that the risk of developing a second malignancy is much higher for AYA patients than for children with cancer or older cancer patients [11,15]. Survivors of tumors, such as breast carcinoma or testicular carcinoma—two of the most common tumor entities in young adulthood—are at increased risk of developing second malignancies [16]. Due to often aggressive and intensive therapies, AYA patients face a multitude of treatment-related side effects and long-term consequences. Accordingly, FCR is a realistic fear that is to a certain degree considered normal for cancer survivors. However, in addition to their complex developmental tasks, young cancer patients are a distinct and vulnerable population who report higher psychosocial distress than their healthy peers, mainly due to unmet information and support needs and a lack of social participation [17,18].

Research on the psychological well-being of AYAs has found that one in four young adults with cancer report increased psychological distress, and symptoms of anxiety, depression, and post-traumatic stress disorder are the most common comorbidities for this group [19,20,21]. Several studies have found that increased FCR is strongly associated with younger age [22,23,24]. A recent meta-analysis supports this significant association, albeit with small effects [25], which indicates a high prevalence of FCR in the AYA group. In several studies, problematic and high prevalence rates have been observed for the majority (>60%) of AYA study participants [26,27,28]. In contrast, Sun and colleagues [29] and Cho and colleagues [30] identified high FCR in 35.74% and 13% of young cancer patients, respectively. In a review by Yang and colleagues [31], FCR prevalence rates among young cancer patients ranged from 31 to 85.2%. A review by Simard and colleagues [9] found clinically relevant FCR in 0–15% of cancer patients of all ages and moderate to high FCR in 22–87% of study participants. These varying prevalence rates are in part due to disagreement in the literature regarding a clinically relevant cut-off for FCR [32,33]. Moreover, several conceptual models exist for FCR and its symptomatology and associations with other constructs. The lack of consensus on a definition of FCR and the resulting lack of agreement on a conceptual model have led to a multitude of divergent measurement instruments [34]. These measurement instruments include different items that vary in sensitivity and ability to capture the extent of FCR. As a result, the differing prevalence rates in the literature are likely based on different measurement criteria.

Uncertainties persist regarding the underlying mechanisms of action of FCR [35]. Identification of such mechanisms is essential for the development of targeted interventions to reduce FCR and to help patients avoid long-term psychological consequences and improve their quality of life, considering the specific needs of different age groups. Adequate interventions do not yet exist specifically for the AYA group [36,37].

To date, there is a gap in the literature regarding the components of FCR and their relationships with each other. Recently, network modeling has been applied in the field of questionnaires to precisely identify the unique relationships between symptoms, which enables researchers to capture complex interactions as a reflection of the overall construct of a questionnaire [38,39]. In the context of the present study, a network modeling approach means that individual items describe general dependency structures that, taken together, contribute to FCR through their connections and interactions. For example, one symptom may lead directly to another symptom, depending on the position of the items in the network [40].

The aim of the current study is to model the individual symptoms of FCR in young cancer patients using a standardized questionnaire as a network, including centrality and predictability of symptoms. The Fear of Progression Questionnaire (FoP-Q-12) was chosen because it represents the gold standard in Germany and has meanwhile been validated in several samples and different languages. It achieved very good quality criteria. In terms of content, the FoP covers the key attributes of FCR and captures not only emotional state but also the associated life domains such as occupation or family. Furthermore, the questionnaire addresses cancer survivors and patients with an acute disease at the same time, as the items measure not only the recurrence but also the progression of a cancer disease.

The following questions will be investigated in the study:

- What is the prevalence of FCR in a German sample of young adult cancer patients?
- What are the most important symptoms in the network of FCR symptoms?
- How are these symptoms related to one another?

The study findings will provide insight into the structure of the questionnaire items and the frequency and severity of individual symptoms of FCR in the AYA group, which will support the development of targeted interventions for this group.

## 2. Materials and Methods

### 2.1. Data Collection

A web-based cross-sectional survey was administered from November 2017 to February 2018 with the following eligibility criteria: (1) cancer diagnosis in the past 10 years; (2) age between 15 and 39 years at the time of survey completion; and (3) having finished acute cancer treatment. Due to a lack of international consensus on the age definition of AYAs, an age range from 15 to 39 years was selected, according to the National Comprehensive Cancer Network (NCCN) definition (Coccia, 2019). The online questionnaire was primarily distributed through social media (e.g., Facebook). A call for study participation with a link to the survey was also published in forums and newsletters of cancer and AYA support groups. In addition, information about the study was provided to patients by the medical staff at the oncological and pediatric oncological wards of the Leipzig University Hospital and the University Hospital Jena.

The survey was made accessible online or a paper-and-pencil questionnaire was sent to the patient on demand. The survey was anonymous, and the disclosure of sensitive personal information was not required. Participants using the hard copy version of the questionnaire provided written informed consent. In completing the online survey, participants provided informed consent by clicking the “I agree” button on the first page of the survey.

### 2.2. Measures

The short version of the Fear of Progression Questionnaire (FoP-Q-12; Table S1) was used to assess FCR [41]. The validated questionnaire contains 12 items measured on a 5-point Likert scale (1 = “never” to 5 = “very often”). Items were aggregated to a summary score ranging from 12 to 60, for which higher values indicated higher levels of FCR. The internal consistency in the sample was α = 0.86. This questionnaire has been used in several studies and is considered a valid and reliable measurement [22,42,43,44]. According to Sarkar and colleagues [45] and Herschbach [46], a cut-off score of ≥34 was used to distinguish between functional and dysfunctional levels of FCR as suggested in most studies. Additionally, participant sociodemographic data (i.e., age, gender, marital status, children, education, and income) and medical information (i.e., diagnosis, time since diagnosis, treatment, and comorbidities) were obtained via self-report.

### 2.3. Statistical Analysis

Data were analyzed using SPSS 27.0 (IBM Corp. Released 2021. IBM SPSS Statistics for Windows, Version 27.0. Armonk, NY: IBM Corp). Descriptive statistics were calculated for all variables and presented as frequencies, mean, median, and standard deviation/interquartile range. Estimation, visualization, and evaluation of the network analysis were conducted using R version 4.1.0 and the RStudio environment.

The package *haven* was used to read the SPSS file and the package *dplyr* was used to rename relevant variables [47]. The R package *qgraph* [48] was applied to estimate and visualize the network with the Fruchterman–Reingold algorithm and the layout *spring*. Spearman’s correlation matrices were calculated and displayed in a network analysis as partial correlations. The nodes in the network represent the different items of the FoP questionnaire. The edges are the lines connecting the nodes and represent the partial correlation coefficients between the items. Thus, an existing edge between two nodes can be interpreted as a present relation between the two variables, considering all other nodes in the network. A thicker edge indicates a stronger underlying association between the variables. Highly correlated nodes appear closely together in the figure, and nodes mapped closer to the center have more correlations with other items than those mapped farther from the center.

We used the least absolute shrinkage and selection procedure (LASSO) to shrink small edges to exactly zero and avoided false-positive edges by dropping them from the model as a regularization technique [49]. To achieve the best fitting network, the extended Bayesian information criterion (EBIC) was minimized with the tuning parameter γ [50].

After the network estimation, we calculated the centrality measure *strength* to gain insight regarding the structural relationships of the network [40]. Strength defines the sum of the absolute weights of edges per node, which indicates the strength of a node’s connection to the other nodes in the network. Two other indices (*betweenness* and *closeness*) exist in network analysis; however, neither is recommended for use in psychological research due to the complexity of their assumptions [51]. The stability of strength was measured using the correlation stability (CS) coefficient. This coefficient specifies the maximum percentage of cases that can be omitted while maintaining a 95% probability that the correlation between strength of the initial sample and bootstrapped subsets is at least 0.7 [38]. CS coefficients range from 0 to 1; for the purposes of this study, the CS coefficient should not be lower than 0.25 and a coefficient greater than 0.5 is considered ideal. Bootstrapped difference tests were conducted with the package *bootnet* to test the accuracy of the network, in accordance with Eskamp [38]. These tests indicate whether one edge weight differs significantly from another.

## 3. Results

### 3.1. Participants

The study sample included 247 participants aged 17–39 years. Participants’ sociodemographic and medical information are summarized in Table 1. The median age was 31 years (interquartile range: 9). The majority of participants were female (82%) and not married (70%). Most participants had completed chemotherapy (81%) and surgery (75%). Most participants had previously been diagnosed with a hematological malignancy (32%), followed by breast cancer (28%) or gynecological cancers (12%). The median time since cancer diagnosis was 35 months (interquartile range: 41).

### 3.2. Prevalence of FCR

The mean score of FCR in the study sample was 35.9 (SD = 9.9), with a range of 12–60. The majority of patients (59.5%) had high FCR according to the cut-off of ≥34. The item with the highest mean was “Being nervous prior to doctor’s appointments or periodic examinations” (M = 3.7), and the item with the lowest mean was “Being afraid by the possibility that the children could contract cancer“ (M = 2.5). Means and standard deviations of all items are displayed in Table S1 in the Supplemental Material.

### 3.3. Network Estimation

Table S1 in the Supplemental Material shows the calculated regularized partial correlation network with the 12 variables of the FoP-Q-SF conducted among an overall sample of 247 participants. Each node represents a single questionnaire item; thus, all nodes have a positive correlation.

**Figure 1:**
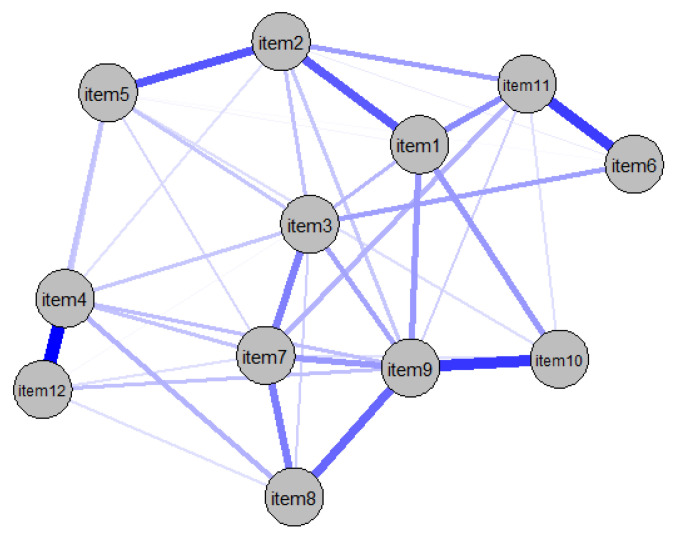
Partial correlation network of FoP-SF items.

Of the 66 possible edges, 41 were significant because they had an absolute weight greater than zero. As illustrated in Figure 1, the relationship between item 4 (“Being afraid of becoming less productive at work”) and item 12 (“Being afraid of not being able to work anymore”) was stronger than 36 of the 41 significant edges. The link between items 9 (“Being afraid of severe medical treatments in course of illness”) and 10 (“Worrying that medications could damage the body”) was also very strong. Weak connections were identified between items 11 (“Worrying what will become of family if something happens to me”) and 12 (“Being afraid of not being able to work anymore”) and between items 5 (“Having physical symptoms, e.g., rapid heartbeat, stomachache”) and 6 (“Being afraid by the possibility that the children could contract the disease”). For a more detailed network visualization, see Figure S2 in the Supplemental Material.

To test the accuracy of the network, the bootstrapped confidence intervals of the edge weights were examined. Most of the edge weights did not differ significantly from the other edges (Figure S1). However, some of the confidence intervals were larger than 0; therefore, the order of the edges in the network should be interpreted with caution. In addition to investigating the accuracy of the edge weights, significance tests were conducted to determine whether one edge is significantly stronger than another (Figure S2). The edges between items 4 and 12, items 9 and 10, and items 6 and 11 were significantly stronger than 50% of the other network edges.

### 3.4. Network Centrality and Stability

**Figure 2:**
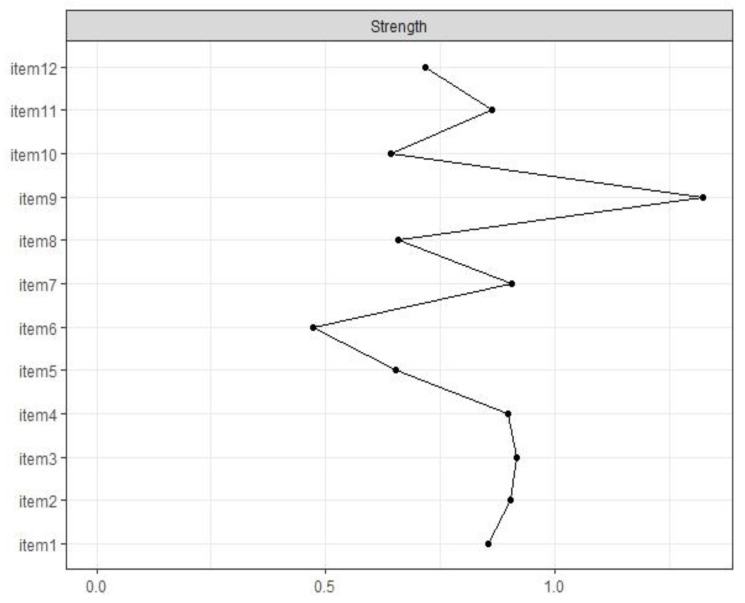
Standardized centrality measure for strength of all items of the FoP-SF.

The standardized centrality index for strength is visualized in Figure 2, which indicates the importance of the items within the network. The items with the highest node strength were items 3 (“Being afraid of pain”), 7 (“Being afraid of relying on strangers for activities of daily living”), and 9. Item 6 was the least central node of all items, as is visually mapped in the network (see Figure 1).

Significance tests confirmed that item 9 was the most central node of all of the items. Additionally, item 6 was significantly less central than all other items except four other variables (Figure S3). Stability analyses revealed a stable network with a CS coefficient of 0.51. Based on this finding, the network can be defined as stable based on the high stability cut-off of 0.5.

## 4. Discussion

### 4.1. General Results

Living with a life-threatening disease such as cancer causes uncertainty and fear of the future for many patients. Cancer patients must deal with possible complications, recurrence, or progression of the disease, as well as physical and psychological limitations, including fear of death. For this reason, FCR is one of the most common concerns among cancer survivors.

The majority of studies on associations and predictors of FCR have confirmed that AYA patients suffer from FCR more often than cancer patients over 40 years of age [28,52]. Prevalence estimates of FCR in the AYA cancer patient group range from 13 to 85% [30,31]. The prevalence estimate of the present study falls within this range. The majority (59%) of study participants revealed dysfunctional FCR, in accordance with the findings of Lane and colleagues [26]. The reasons for heightened FCR among young patients have not yet been definitively identified. Researchers suspect that these reasons include heightened psychological stress, lowered resilience, and concern regarding upcoming developmental tasks (e.g., having one’s own children or meeting occupational commitments) among patients in this age group, as well as the perception of being too young for cancer [9,36,53].

Men were underrepresented in the sample, which is certainly the case in many psycho-oncological studies [54]. One possible explanation is that women are generally more open to the topic and more willing to participate in a study that answers questions about their psychological well-being [55]. In addition, different levels of stress or differences in coping strategies between women and men could explain the differences in participation rates. In addition, the recruitment process (e.g., via social media) may have contributed to the gender imbalance. This gender imbalance could, in turn, explain why the majority of study participants had high FCR levels. This is because women generally have a higher lifetime prevalence of developing an anxiety disorder than men [56]. Similarly, a recent review showed that women with cancer report higher FCR than male cancer patients [57].

Another important driver of FCR may relate to the different coping strategies used by patients in different age groups [58]; specifically, older people are better at regulating their emotions than younger people. According to studies, AYAs report a number of unmet needs regarding appropriate coping strategies to manage FCR [59]. Persistently high FCR among this group increases their risk of psychiatric comorbidities, reduced quality of life, and detrimental health behaviors such as avoidance of follow-up visits (or, conversely, high-frequency physician visits) or excessive self-examinations [60,61]. Current research findings suggest that FCR remains relatively stable during survivorship. Due to the longer lifetime and subsequent extended survivorship phase of AYA patients, the investigation of the underlying mechanisms of FCR in this group is warranted [52,62]. The present study used network analysis to precisely examine the complexity of FCR based on a questionnaire and its individual items.

First, the network of FCR in the FoP-SF questionnaire can be considered a stable network based on the CS coefficient and high bivariate correlations. The original version of the questionnaire consists of five dimensions, four of which are affirmed in the present network (i.e., items of each dimension are neighboring nodes that are significantly related to each other), even though the short form of the questionnaire is unidimensional. This finding confirms the validity of the FoP-SF instrument and indicates that it could be used as a standard in future studies. Nevertheless, an appropriate cut-off score must be defined. Currently, differing cut-off values are used for the FoP-SF and for other questionnaires on FCR, as mentioned above [44,45].

Another important result is related to the centrality measures, which can be used to determine which items play a central role in the network and may activate other items. The decentrality of item 6 may result from a sample bias. More than 70% of the study participants did not (yet) have children of their own, so item 6 might therefore be less relevant and therefore less central for them than it would be among a sample of parents. In the network, item 9 (“Being afraid of severe medical treatments in course of the illness”) played a central role in understanding FCR, with a significant standardized node strength centrality of 1.30. Further, this item had the largest number of significant connections to other items, with particularly strong connections to neighboring items 8 (“Being afraid of no longer being able to pursue hobbies”) and 10 (“Worrying that medication could damage the body”). This finding could be interpreted to indicate that patients are afraid that severe medical interventions and medication could damage their bodies to an extent that would prevent them from organizing their daily lives in a self-determined way. Finally, the development and maintenance of autonomy is an important focus of young adulthood [63,64]; thus, an alternative interpretation of this finding could be that severe medical interventions indicate to patients that their disease is particularly severe and trigger thoughts of possible mortality, which in turn increase anxiety.

Another explanation for the centrality of item 10 could be that young cancer patients are uncertain about what to expect from severe medical interventions, possibly due to a lack of health literacy or information about such interventions. Studies have found that AYA patients report a wide range of unmet information needs [65,66]; however, the existing research on health literacy in this group is insufficient to draw a strong conclusion [67,68]. Several studies have demonstrated an association between unmet information needs and heightened FCR [1,9,69,70]. Although the internet and social platforms are important integral parts of young cancer patients’ information seeking, these tools do not replace direct physician-patient conversations about the individual’s disease and treatment [71,72,73]. Medical staff should regularly monitor patient psychological distress, including FCR, throughout the course of treatment and follow-up and provide adequate support services as needed [52]. Oncologists can specifically address patient uncertainties and provide detailed education about oncological treatments. These discussions could also potentially alleviate patients’ fear of severe medical procedures, which in turn could avoid triggering additional FCR symptoms. Further research is required to identify and understand dynamics of the communication process that should be considered in future interventions with cancer survivors and their healthcare providers to ensure that all information needs are met.

### 4.2. Strengths and Limitations

To our knowledge, this is the first study to examine the construct of FCR using network analysis. The strengths of the study include the large sample of young cancer patients and the estimation of a stable network of the standardized and validated FoP-SF questionnaire.

Nevertheless, the study must be interpreted in accordance with its limitations. The gender distribution in the sample was characterized by a high proportion of women, which could have led to a bias. Additionally, the study design did not enable us to determine whether the most central symptom is responsible for the activation of other symptoms or is caused by other symptoms. Further, this is a cross-sectional study; thus, causal and temporal inferences are not possible. Further network analyses among patients of other age groups with a balanced gender distribution are needed for comparison to verify whether the item structure can be replicated or whether it is a phenomenon specific to the AYA patient group. Longitudinal studies could reveal changes in symptomatology over time, which could lead to conclusions about conditionality and the interactions between individual symptoms.

## 5. Conclusions

Cancer diagnosis in young adulthood is associated with increased fear of cancer recurrence. Accordingly, the need for age-specific support services is strong. Thus far, the construct of FCR—including its predictors, consequences, and symptoms—has not been fully elucidated. For the first time, a network analysis was applied to model the structure of an FCR questionnaire. Based on the study findings, conclusions can be drawn regarding which symptoms of FCR play a central role for young adult cancer patients. The results indicate that symptoms of FCR should be used to inform the development of tailored interventions that target the most important issues for patients (e.g., maintaining autonomy, acquiring adaptive coping strategies, improving physician-patient communication) and to maximize the benefit of individual therapies.

## Figures and Tables

**Table 1:** Sample demographic and medical characteristics (*n* = 247).

Variables		*n*	% ^1^
Age in years (median (interquartile range))		(31.0 [9])	
Gender	Female	202	81.8
	Male	45	18.2
Partnership/Cohabiting (yes)		160	64.8
Children (yes)		67	27.1
Educational level	Junior high school or below	64	25.9
	High school degree	178	72.1
Employment status	Employed	174	70.4
	Unemployed	53	21.5
	Students	20	8.1
Off treatment (yes)		213	86.2
Comorbidities (yes)		130	52.6
Tumor site	Hematological malignancies	78	31.6
	Solid tumors Breast cancer Gynecological cancer Sarcoma Other	- 70 30 19 50	- 28.3 12.1 7.7 20.2
Time since diagnosis in months (median (interquartile range))		(35.2 [41.0])	
Cancer treatments	Surgery	185	74.9
(multiple responses possible)	Radiation therapy	127	51.4
	Chemotherapy	200	81.0
	Bone marrow/stem cell transplantation	14	5.7
	Hormonal therapy	46	18.6

^1^ Percentages may not add up to 100 due to missing data.

## Data Availability

The data presented in this study are not publicly available due to privacy or ethical restrictions. Requests to access these datasets should be directed to the corresponding author.

## Supplementary Materials

The following resources are available online at https://www.mdpi.com/article/10.3390/cancers14092092/s1, Table S1: Mean scores and standard deviations of the FoP-Q-12 items; Table S2: Regularized partial correlation coefficients of the edges; Figure S1: Bootstrapped confidence intervals of estimated edge-weights; Figure S2: Bootstrapped stability test for edge-weights; Figure S3: Standardized node strength centrality for FoP-SF items.
