# Supplementary material for: Fear of Recurrence in Young Adult Cancer Patients—A Network Analysis
Source: Cancers (Basel). 2022 Apr 22;14(9):2092. doi: 10.3390/cancers14092092 (PMC9105535; doi:10.3390/cancers14092092)
Supplement: Supplementary file 1 [file cancers-14-02092-s001.zip › cancers-1596213-supplementary.pdf]

cancers

MDPI

*Supplementary Materials*

# Fear of Recurrence in Young Adult Cancer Patients—A Network Analysis

**Diana Richter, Katharina Clever, Anja Mehnert-Theuerkauf and Antje Schönfelder**

**Table S1.** Mean scores and standard deviations of the FoP-Q-12 items.

| Item | Mean | SD |
|---|---|---|
| 1. Being afraid of disease progression | 3.14 | 1.1 |
| 2. Being nervous prior to doctors' appointments or periodic examinations | 3.68 | 1.29 |
| 3. Being afraid of pain | 2.71 | 1.30 |
| 4. Being afraid of becoming less productive at work | 3.17 | 1.28 |
| 5. Having physical symptoms, e.g., rapid heartbeat, stomach ache, nervousness | 3.45 | 1.26 |
| 6. Being afraid by the possibility that the children could contract cancer | 2.49 | 1.51 |
| 7. Being afraid of relying on strangers for activities of daily living | 2.66 | 1.33 |
| 8. Being afraid of no longer be able to pursue hobbies | 2.60 | 1.28 |
| 9. Being afraid of severe medical treatments in course of the illness | 2.98 | 1.25 |
| 10. Worrying that medications could damage the body | 3.10 | 1.38 |
| 11. Worrying about what will become of the family if something should happen to the patient | 3.21 | 1.37 |
| 12. Being afraid of not being able to work anymore | 2.70 | 1.40 |

SD: standard deviation.

**Table S2.** Regularized partial correlation coefficients of the edges.

| Item | 1 | 2 | 3 | 4 | 5 | 6 | 7 | 8 | 9 | 10 | 11 | 12 |
|---|---|---|---|---|---|---|---|---|---|---|---|---|
| **1** | | | | | | | | | | | | |
| **2** | **0.25*** | | | | | | | | | | | |
| **3** | **0.11*** | **0.09*** | | | | | | | | | | |
| **4** | 0 | **0.05*** | **0.08*** | | | | | | | | | |
| **5** | **0.02*** | **0.25*** | **0.09*** | **0.08*** | | | | | | | | |
| **6** | 0 | **0.03*** | **0.14*** | 0 | **0.01*** | | | | | | | |
| **7** | 0 | 0 | **0.19*** | **0.09*** | **0.06*** | 0 | | | | | | |
| **8** | 0 | 0 | **0.07*** | **0.11*** | 0 | 0 | **0.20*** | | | | | |
| **9** | **0.15*** | **0.09*** | **0.14*** | **0.10*** | 0 | 0 | **0.15*** | **0.23*** | | | | |
| **10** | **0.15*** | 0 | 0 | 0 | **0.05*** | 0 | **0.05*** | 0 | **0.30*** | | | |
| **11** | **0.17*** | **0.15*** | 0 | 0 | 0 | **0.29*** | **0.12*** | 0 | **0.07*** | **0.05*** | | |
| **12** | 0 | 0 | 0 | **0.38*** | **0.09*** | 0 | **0.05*** | **0.05*** | **0.10*** | **0.04*** | **0.01*** | |

* $p < 0.05$.

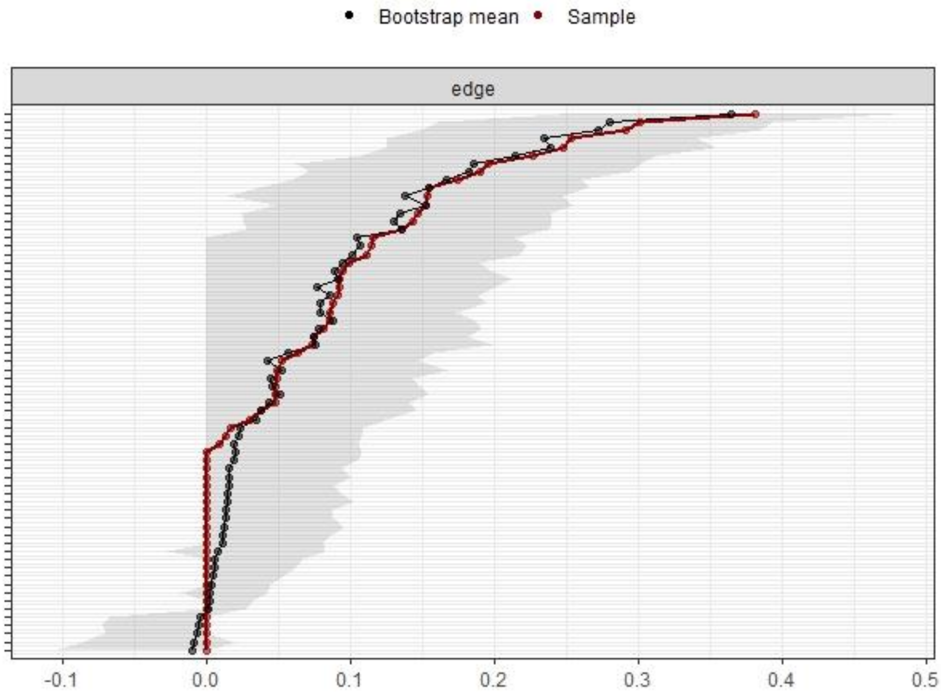


**Figure S1.** Bootstrapped confidence intervals of estimated edge-weights for the estimated network of FoP-SF items. The red line shows the sample values and the gray area the bootstrapped confidence intervals. Each horizontal line represents one edge. Smaller confidence intervals indicate more accurate edge estimates.

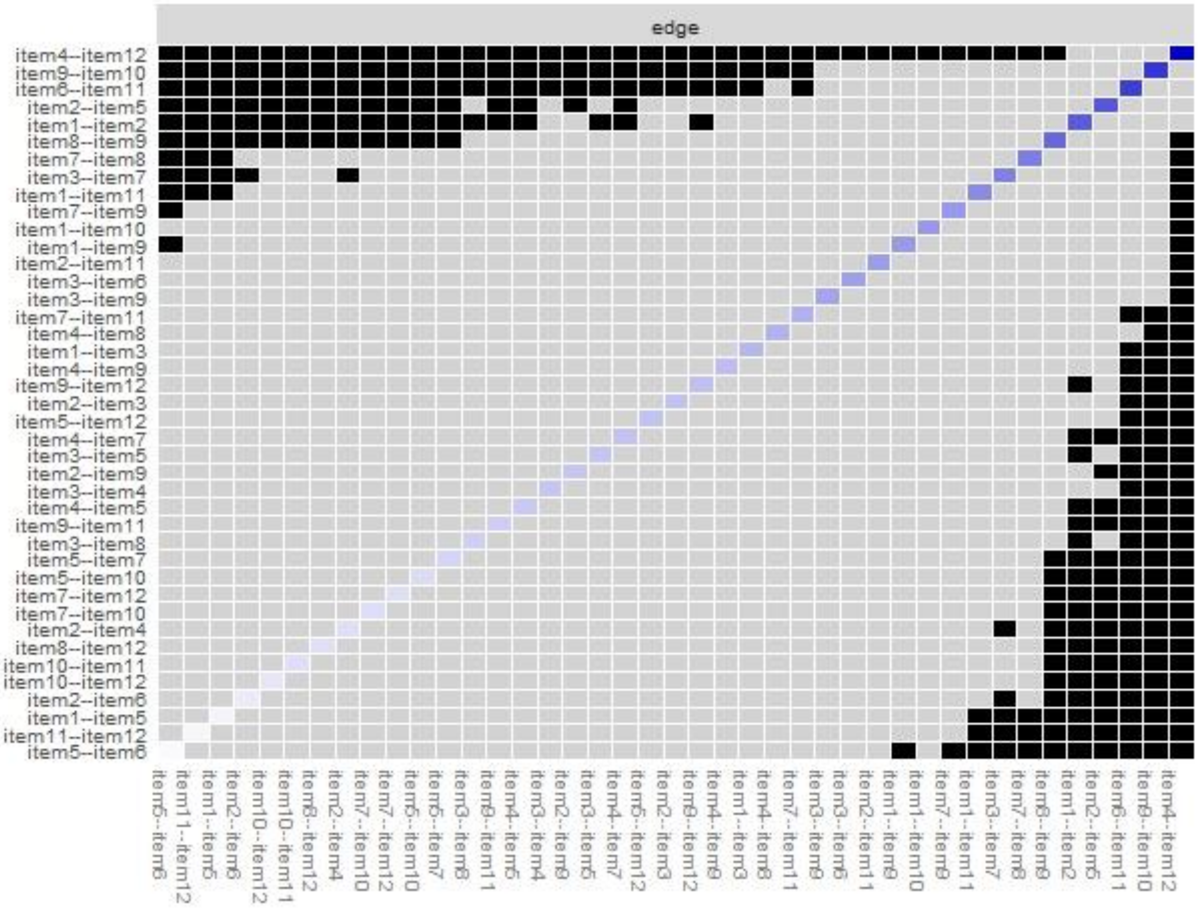


**Figure S2.** Bootstrapped stability test for edge-weights. The color of the boxes shows whether edge-weights differ significantly from each other (i.e., black) or do not differ significantly (i.e., grey). The diagonal line indicates the strength of edge-weights, shifting from blue ((representing stronger edges), to white (representing weaker edges).

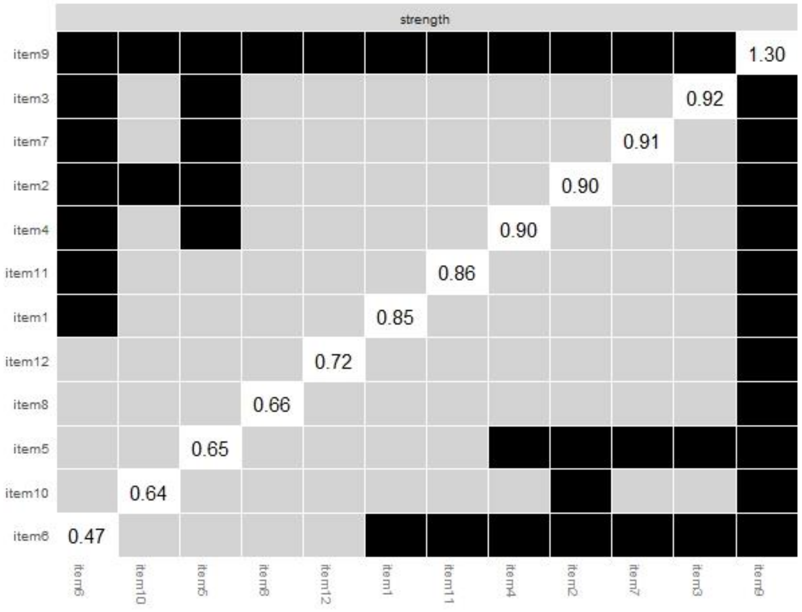


**Figure S3.** Standardized node strength centrality for FoP-SF items. Black boxes represents a significant difference in node strength for each pairing, grey boxes a non-significant difference. The number in the white boxes (diagonal line) shows the value of node strength of a specific node.
